# Genotypic traits and tradeoffs of fast growth in silver birch, a pioneer tree

**DOI:** 10.1007/s00442-021-04986-9

**Published:** 2021-07-26

**Authors:** Juha Mikola, Katariina Koikkalainen, Mira Rasehorn, Tarja Silfver, Ulla Paaso, Matti Rousi

**Affiliations:** 1 Faculty of Biological and Environmental Sciences, Ecosystems and Environment Research Programme, University of Helsinki, Niemenkatu 73, 15140 Lahti, Finland; 2 Natural Resources Institute Finland (Luke), Latokartanonkaari 9, 00790 Helsinki, Finland; 3 Department of Environmental and Biological Sciences, University of Eastern Finland, P.O. Box 1627, 70211 Kuopio, Finland; 4 Present Address: Ramboll Finland, Niemenkatu 73, 15240 Lahti, Finland

**Keywords:** Allocation cost, *Betula*, EM fungi, Herbivory, Resource competition

## Abstract

Fast-growing and slow-growing plant species are suggested to show integrated economics spectrums and the tradeoffs of fast growth are predicted to emerge as susceptibility to herbivory and resource competition. We tested if these predictions also hold for fast-growing and slow-growing genotypes within a silver birch, *Betula pendula* population. We exposed cloned saplings of 17 genotypes with slow, medium or fast height growth to reduced insect herbivory, using an insecticide, and to increasing resource competition, using naturally varying field plot grass cover. We measured shoot and root growth, ectomycorrhizal (EM) fungal production using ergosterol analysis and soil N transfer to leaves using ^15^N-labelled pulse of NH_4_^+^. We found that fast-growing genotypes grew on average 78% faster, produced 56% and 16% more leaf mass and ergosterol, and showed 78% higher leaf N uptake than slow-growing genotypes. The insecticide decreased leaf damage by 83% and increased shoot growth, leaf growth and leaf N uptake by 38%, 52% and 76%, without differences between the responses of fast-growing and slow-growing genotypes, whereas root mass decreased with increasing grass cover. Shoot and leaf growth of fast-growing genotypes decreased and EM fungal production of slow-growing genotypes increased with increasing grass cover. Our results suggest that fast growth is genotypically associated with higher allocation to EM fungi, better soil N capture and greater leaf production, and that the tradeoff of fast growth is sensitivity to competition, but not to insect herbivory. EM fungi may have a dual role: to support growth of fast-growing genotypes under low grass competition and to maintain growth of slow-growing genotypes under intensifying competition.

**Supplementary Information:**

The online version contains supplementary material available at 10.1007/s00442-021-04986-9.

## Introduction

Much of the theory and empirical research of the ecology of plant communities and populations is built on tradeoffs between plant traits (Coley et al. 1985; Grime 2001; Rees et al. 2001; Craine 2005; Viola et al. 2010; Agrawal 2011; Antonelli et al. 2011; Kempel et al. 2011; Lind et al. 2013). Tradeoffs arise from allocation costs, i.e. when allocation of a common, limited resource to one trait (e.g. herbivore defense) decreases its allocation to another trait (e.g. growth). According to the theory of plant successional dynamics, early-successional pioneer species are fast growers when resources are abundant, but poor competitors when resources get scarce (Rees et al. 2001), i.e. they have a tradeoff between growth rate and competitive ability. Therefore, although fast-growing species have high leaf nitrogen (N) concentrations and high carbon (C) acquisition (Wright et al. 2004), they can only persist by colonizing disturbed sites earlier than late-successional dominant competitors (Rees et al. 2001). Another theory suggests that fast growth is linked to low herbivore resistance because growth and herbivore defense compete for common resources (Herms and Mattson 1992; Fine et al. 2004, 2006) and because generalist herbivores prefer fast-growing, early-successional species that invest less to herbivore defense than late-successional species (Coley et al. 1985; Fraser and Grime 1999). In plants, the cost of fast growth is thus predicted to emerge as a susceptibility to herbivory and resource competition.

Despite the clean logic of plant economic tradeoffs, their detection remains challenging (Agrawal 2011). In an early meta-analysis, Koricheva (2002) found no common trend of tradeoffs between measures of herbivore defense and plant fitness, but instead, allocation costs of defense varied with the environmental setting of a study. Growing conditions with multiple herbivores, competitors and abiotic stress appeared best in revealing the tradeoffs (Koricheva 2002). A literature review by Strauss et al. (2002) suggested that costs of herbivore defense are widespread, but also this assessment suggested that allocation costs may appear through interactions with co-occurring organisms, such as increasing resistance to herbivores decreasing resistance to pathogens or lessening attractiveness to mutualists. That some of the most convincing evidence of tradeoffs between herbivore resistance and growth rate has since been found in experiments in natural field settings (Fine et al. 2004, 2006) further supports the impression that natural context with diverse species interactions can be important for detecting tradeoffs.

Many seminal publications of plant tradeoffs focus on aboveground traits only, but several studies have also searched for correlations between aboveground and belowground plant traits (Craine and Lee 2003; Craine et al. 2005; Tjoelker et al. 2005). It has been suggested that a parallel division between fast-growing and slow-growing species exists in root traits as is found for shoot traits (Reich 2014), but this proposition has also been questioned (Kong et al. 2016, 2019). An essential part of root and plant ecology is their symbiotic relationships with soil microorganisms (Bever et al. 2010; Bardgett et al. 2014; De Deyn 2017). Mycorrhizal fungi are most numerous and abundant of the symbiotic partners, with over 80% of terrestrial vascular plant species forming a relationship with mycorrhizal fungi (Smith and Read 2008; Brundrett 2009). The key role of mycorrhizal fungi in controlling plant traits and community dynamics is shown by their ability to influence the global biogeography (Delavaux et al. 2019) and productivity and diversity (van der Heijden et al. 1998) of plants. Of the mycorrhizal associations, the ectomycorrhizal (EM) and arbuscular mycorrhizal (AM) associations are the most widespread, and while they differ in fungal phyla and structure, as well as the associated plant families, they serve similar functions to their host plants. In the symbiosis, the fungal partner enhances plant uptake of nutrients and water in exchange for carbohydrates produced in photosynthesis (Smith and Read 2008), but the fungi can also play a role in plant tradeoffs: they can affect plant competitive interactions (Bever et al. 2010; Stanescu and Maherali 2017) and herbivore resistance (Koricheva et al. 2009; Vannette and Hunter 2013) and can themselves depend on plant traits such as herbivore resistance (Sthultz et al. 2009). In general, fast-growing species growing in soils with high availability of mineral nutrients are predicted to allocate less resources to belowground mutualists than slow-growing species that typically occur in soils with nutrient deficiency (De Deyn 2017). However, while there is a growing recognition of the importance of including roots and belowground interactions in considerations of plant community dynamics and tradeoffs (Bever et al. 2010; Mommer and Weemstra 2012; Bardgett et al. 2014; De Deyn 2017), the empirical work is still lagging far behind the aboveground research.

One of the main consequences of tradeoffs of plant traits in plant communities is the maintenance of species diversity (Grime 2001; Rees et al. 2001; Viola et al. 2010). At the population level, tradeoffs are equally needed to explain why natural selection does not erode intrapopulation genetic variation in plant traits. Here, we present results from a field study, where we searched for genotypic traits and tradeoffs of fast growth in a local silver birch, *Betula pendula* population by exposing genotypes with slow, medium and fast growth (sensu Reich 2014) to experimentally manipulated insect herbivory and naturally varying grass cover. Silver birch is one of the most common broad-leaved tree species in Europe (Atkinson 1992) and has become a boreal model species for tree genome analysis (Salojärvi et al. 2017). It is a typical early-successional plant with abundant production of light-weight seeds (Rousi et al. 2019) and an ability to rapidly colonize disturbed forest patches (Atkinson 1992; Fischer et al. 2002). It also has the typical tradeoffs of a pioneer species. It is fast-growing in fertile sites, but seedling growth is sensitive to resource competition by herbaceous vegetation (Ferm et al. 1994; Mikola et al. 2014) and herbivory can significantly affect the survival and growth of the seedlings (Prittinen et al. 2003; Silfver et al. 2013, 2015). Similar to many temperate and boreal forest trees, silver birch forms a symbiosis with EM fungi, with clear positive effects on seedling growth across various soils (Frankland and Harrison 1985). In contrast to aboveground traits, however, little is known of the genetic variation in EM association or the genetic links between EM colonization and aboveground traits.

Silver birch is a wind-pollinated species with great pollen production and dispersal (Siljamo et al. 2008; Geburek et al. 2012; Rousi et al. 2019). The efficient pollen transfer mixes the genetic materials that are under selection in local populations and thus, helps in maintaining the intrapopulation genetic variation (Stener and Hedenberg 2003; Stener and Jansson 2005). We have earlier shown that growth differences among genotypes in our study population can be 3.5- to 5.5-fold, with the coefficient of genotypic variation in the range of 0.21–0.37 (Mikola et al. 2014). In the present study, we first examined, with an emphasis on root growth, soil nutrient acquisition and EM symbiosis, if the fast-growing and slow-growing genotypes of this population exhibit the typical traits of fast-growing and slow-growing plant species (Wright et al. 2004; Reich 2014; De Deyn 2017). We hypothesized that fast-growing genotypes (defined by their fast shoot elongation) have greater production of leaves of higher N concentration, greater root production and better capture of soil N pulses, but lower EM fungal production and lower root mass–leaf mass ratio to signify smaller relative allocation to belowground than aboveground resource acquisition. Second, we tested if the typical tradeoffs of fast growth (Coley et al. 1985; Herms and Mattson 1992; Rees et al. 2001) emerge among the genotypes. We hypothesized that shoot elongation, leaf production and soil N capture will be more adversely affected by insect herbivory and resource competition for fast-growing than slow-growing genotypes. If supported, these predictions would first suggest that tradeoffs of fast growth also emerge at intrapopulation level and second, that through preventing selection solely favoring fast growth, the tradeoffs may significantly contribute to the genetic variation found in local silver birch populations.

## Materials and methods

### Field site and plant material

The experimental field site is situated in Loppi, south Finland (60° 36′ N, 24° 24′ E), where the thermal growing season (mean daily temperature > 5 °C) starts at the end of April and ends in the middle of October and where the mean annual precipitation is 660 mm (Finnish Meteorological Institute, 2012). The soil in the site is post-glacial sorted fine sand and the top 5 cm soil layer has a 15% loss on ignition, total C and N concentrations of 6% and 0.3%, respectively, and a pH of 5.0 (Mikola et al. 2014). Before the present study, the site was covered by middle-aged forest stands of *B. pendula* and *Pinus sylvestris* with sporadic *Picea abies* trees. The stands were clear cut in winter 2008, and in spring 2009 six field blocks were established, each divided into 132 planting plots (2 m × 2 m in area). The ground layer vegetation in the site is dominated by the fern *Pteridium aquilinum* (mean areal cover 11%), the graminoids *Calamagrostis arundinacea* (43%) and *Deschampsia flexuosa* (13%), and the dwarf shrubs *Vaccinium myrtillus* (2.7%) and *Vaccinium vitis-idea* (4.5%) (Mikola et al. 2014).

The plant material used in the study originates from a 0.9-ha *B. pendula*–*B. pubescens* forest stand, naturally regenerated after 1979 logging in Punkaharju, south-east Finland (61° 48′ N, 29° 18′ E). The mother trees that were selected from the stand for offspring production grow 10–60 m apart. To establish the Loppi experimental site, 800 saplings of 19 mother trees, or genotypes, were propagated using a tissue-culture technique, or micropropagation (Ryynänen and Ryynänen 1986) in early 2008. The plantlets were grown in a nursery for summer 2008 and overwintered in a cold room before being randomly allocated into planting plots in 2009 (Mikola et al. 2014). Of the 19 genotypes, “16” and “24” were excluded as they grew poorly in the nursery and most of their plantlets died in the field (Mikola et al. 2014). Two saplings of each of the remaining 17 genotypes in each replicate block (altogether 204 seedlings) were randomly selected for the present study (Supplementary Fig. 1).

### Insecticide treatment, grass cover variation and ^15^N-labelled soil N pulse

The response of genotypes to herbivory was examined by spraying one sapling of each genotype in each block with 0.1 l of 0.1% solution of synthetic pyrethrin Decis EC25 (Bayer CropScience, Germany) to reduce insect herbivore load (Supplementary Fig. 1), and the other sapling with equal quantity of water to allow natural herbivore colonization (Silfver et al. 2013, 2015). The saplings were sprayed once a week (with a few exceptions due to particularly rainy weather) using two portable garden sprayers (one for water, the other for the insecticide) during the growing seasons 2010 and 2011. While spraying, the saplings were enclosed within a shower cubicle to prevent insecticide wind drift. The applied insecticide dosage does not trigger side effects in *B. pendula* shoot growth, root growth or leaf chemistry and can, therefore, be safely used for reducing herbivore load (Silfver et al. 2013). The intensity of resource competition in planting plots was visually estimated as the areal cover of graminoids (Supplementary Fig. 1). Each plot was classified into one of the six coverage classes: 1–10%, 11–20%, 21–40%, 41–60%, 61–80% or 81–100% of plot area covered (in graphs and statistical analyses, the mean cover was used for each class). Graminoids were chosen to express the varying competitive pressure by herbaceous vegetation due to their high abundance and high spatial variation in the field site (Mikola et al. 2014).

The ability of saplings to capture mineral N in the soil was assessed by adding a small quantity of ^15^N-labelled (NH_4_)_2_SO_4_ (^15^N atom excess 10%) to the uppermost soil layer (0–3 cm depth) under each sapling. A total of 60 mg (^15^NH_4_)_2_SO_4_, dissolved in 120 ml water, was added below each sapling in 10-ml portions placed at four directions 10, 15 and 30 cm apart from the stem. The labelled N was added in mid July 2011, and 30 days later, five to ten random leaves were collected from each sapling for ^15^N and total N analysis. Leaf material was ground in liquid nitrogen, dried (70 °C, 24 h) and transferred to Iso-Analytical Limited, Cheshire, UK (http://www.iso-analytical.co.uk), a commercial laboratory specialized in stable isotope analysis.

### Measuring plant growth, N uptake, leaf damage and mycorrhizal fungal growth

**Fig. 1:**
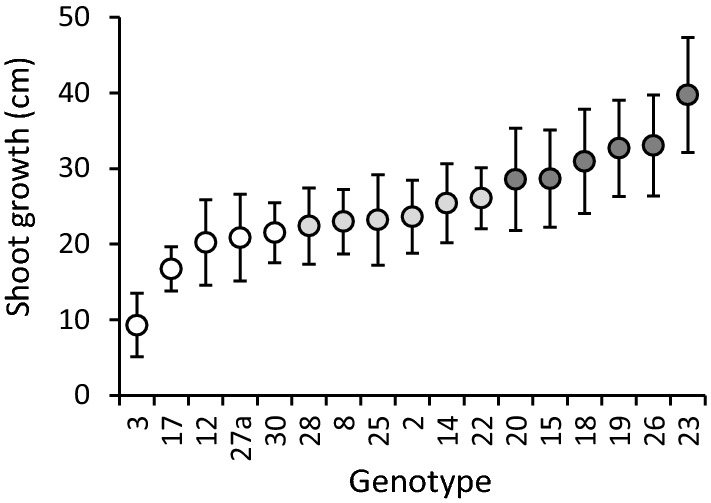
Annual shoot growth (i.e. height increment) of silver birch genotypes (mean ± SE; *n* = 10–12) and their assignment to slow (white), moderate (light grey) and fast (dark grey) growth classes

Birch sapling shoot growth (i.e. height increment) was calculated for year 2011 by measuring the height of each sapling in early spring and late autumn 2011. These growth values were used to assign the genotypes into the three growth classes, and to keep the experimental set-up balanced, a balanced number of genotypes (5, 6 and 6) was allocated into each class (Fig. 1).

Choosing the environmental conditions, where to determine the growth class of a genotype is not a trivial task as the genotype rank in growth (i.e. whether a single genotype appears as fast-growing or slow-growing relative to others) may differ between environments. In particular, it is not appropriate to use nursery growth rates as a proxy for field growth rates as significant genotype × environment (G × E) interactions may occur (Mikola et al. 2014). Therefore, to have growth rate estimates as relevant as possible, we used all 204 saplings (i.e. from both herbivory treatments and along the whole grass cover gradient) to calculate the estimates. In such approach, strong G × E interactions could even out genetic differences, but our earlier results show that the forest ground heterogeneity found at our site does not create strong G × E interactions in silver birch growth (Mikola et al. 2014) and neither is there a genotype × herbivory interaction in our growth data as the rank correlation between genotype means in the control plots and under the insecticide treatment is positive (Spearman’s rho = 0.32).

Leaf number was counted for each sapling and the total leaf dry mass was estimated by multiplying the mean dry mass of leaves collected for N analysis by the number of leaves. Leaf N uptake was calculated using Eq. (1)﻿, where Air ^15^N atom% = 0.3663.

1 $${\text{Leaf}}\,{\text{~N}}\,{\text{~uptake}} = ~\frac{{\left( {{\text{Leaf~}}\,{\text{N}}\% ~ \times ~{\text{Leaf~}}\,{\text{mass}}} \right)~ \times ~({\text{Leaf}}~{}_{{}}^{{15}} {\text{N}}\,{\text{~atom}}\% - {\text{Air~}}{}_{{}}^{{15}} {\text{N}}\,~{\text{atom}}\% )}}{{100~ \times 100~ \times 0.1}}.$$

Leaf damage by herbivores was recorded in late July 2011. For each sapling, the top and the second highest side branch were surveyed and the leaf damage index was calculated using a modified Schreiner-type method (Fritz et al. 1998). In this method, the index of damage is produced using two scores; one gives the average area damaged per leaf (0 = 0%, 1 = 1–4%, 5 = 5–20% and 25 = 21–100% of the leaf area eaten), the other the proportion of the number of leaves damaged (0 = 0%, 1 = 1–25%, 2 = 26–50%, 3 = 51–75% and 4 = 76–100% of the total number of leaves damaged). Multiplication of these two scores gives the damage index that ranges from 0 to 100.

Sapling root growth was estimated using an in-growth core method. A mesh core (depth 10 cm, diameter 5 cm, 6 mm mesh) was installed under two saplings of each of the 17 genotypes in each of the six replicate blocks (total of 204 mesh cores) in June 2010. The core was placed under the longest branch of a sapling at a distance from the stem that was equivalent to a half of the sapling height (we considered that setting the distance from the stem and the size of the sapling in proportion was appropriate as the size of the saplings varied). The mesh core was first installed using a soil corer and a plastic tube (both 5 cm in diameter) and was then filled with root-free, sieved (1 mm mesh) mineral soil, collected from the study site in May 2010. Finally, a layer of humus was placed on the top of the core. The first set of cores was harvested in November 2010 and the second set in November 2011 using a larger soil corer (diameter 9 cm). After collection, the cores were transported to a laboratory and stored at − 20 °C until the roots were washed free of soil over a set of two sieves (1 mm and 0.43 mm mesh). Birch roots were visually sorted out of other roots (the identification was confirmed under a light microscope), freeze-dried and weighed. Too few roots were obtained from the cores harvested in 2010 to reliably estimate root growth, thus growth estimates are based on 2011 cores only. Our original aim was to further characterize root structure by measuring specific root length, surface area, diameter and number of root tips, but the samples turned out to be too few (n = 6 with 2011 samples only) and sparse for reliable estimates for these variables and we chose to stick to a more robust measure of root biomass. We did not make a difference between absorptive and non-absorptive roots. An advantage of low root density also in 2011 cores is that we can assume root growth estimates not to be constrained by the size of the in-growth core. To illustrate relative biomass allocation to roots, an index of root mass–leaf mass ratio was calculated by dividing mesh core root dry mass (mg dm^−3^ soil) by leaf dry mass (g) for each sapling.

In an EM association, the fungus forms three structures: a sheath of fungal tissue that encloses the root, a network of hyphae that grows between plant cells and an outward growing external, or extramatrical mycelium that forms the connection to soil (Smith and Read 2008). We quantified the growth of the external mycelium in the root zone of the saplings using nylon meshbags (10 × 10 × 2 cm, 50 µm mesh; LK-Suodatin Oy, Siivikkala, Finland), filled with quartz sand. The 50 µm mesh size allows for the growth of fungal hyphae, but not that of plant roots (Wallander et al. 2001), and the quartz sand devoid of organic substances should not attract saprotrophic fungi. Prior to meshbag preparation, the quartz sand was ashed (600 °C, 3 h), acid washed and adjusted to pH 4–5 using 1% HCl. The sand was dried at 40 °C for 24 h and a 100-g portion was placed into each meshbag. The bags were sealed using a heat sealer and an electric hot glue gun, and in the field, placed horizontally below the litter layer at the same time and adjacent to the root in-growth cores (one bag for each sapling). The bags were harvested along with root cores, transported to a laboratory and stored at − 20 °C until their ergosterol content was quantified as a proxy of EM fungal mycelium biomass using the method originally described by Nylund and Wallander (1992) and slightly modified by Markkola (1996) and Kasurinen et al. (2001). Prior to ergosterol analysis, the bags were freeze-dried and extracted following the method described in Kasurinen et al. (2001) except that 15 g of dry sand was extracted using 10 ml of 95% ethanol and 2 ml of 60% KOH in demineralised water. For the HPLC analysis, a reverse-phase column (Hewlett-Packard, LiChrospher 100 RP-18) was used with 100% methanol as an eluent. A 20-µl sample was injected and run with 1.6 ml methanol once a minute, and the ergosterol peaks were detected using an UV-detector at 280 nm. In the beginning and at the end of every sample sequence, a set of internal ergosterol standards was run to obtain a regression formula for the standard curve.

### Statistical analyses

The effects of the growth class, grass cover, insecticide treatment and the growth class × grass cover and growth class × insecticide interactions were tested using ANCOVA models. The growth class and insecticide treatment were treated as fixed factors and the grass cover as a covariate. For grass cover, the mean of the lowest and highest cover was used for each cover class (i.e. 5.5, 15.5, 30.5, 50.5, 70.5 and 90.5% of area covered). Although the grass cover variation was not experimentally manipulated, the high number of grass cover measurements (55–74) included in each growth class guarantee a powerful test for the growth class × grass cover interaction. The genotype was included in the models as a random factor nested within the growth class to explain the part of genetic variation that remained within growth classes, and the replicate block was included as a random factor to explain the spatial variation in the field site. As some of the top shoots were damaged, which has an effect on aboveground growth (Mikola et al. 2014), ‘top shoot damage’ was included as a two-level (damaged or not damaged) fixed factor for shoot models, and since ergosterol data was collected in 2010 and 2011, the year was included in the ergosterol model as a fixed factor. The homogeneity of error variances was tested using the Levene’s test and the normality of residual distributions using the Kolmogorov–Smirnov test. To fulfill these criteria, the data of root mass, root allocation index, ergosterol mass and N uptake were square-root-transformed and the data of leaf mass and leaf damage index log-transformed. For these variables, the means and error variation shown in bar charts are back transformed. Finally, for the ergosterol mass–leaf mass ratio, the significance of the difference between the slow growth class and fast growth class was tested using a pairwise comparison of estimated marginal means.

## Results

The genotype means of shoot growth varied from 9.3 to 39.7 cm (Fig. 1). Based on these means, the genotypes were sorted into slow, moderate and fast growth classes (Fig. 1).

**Fig. 2:**
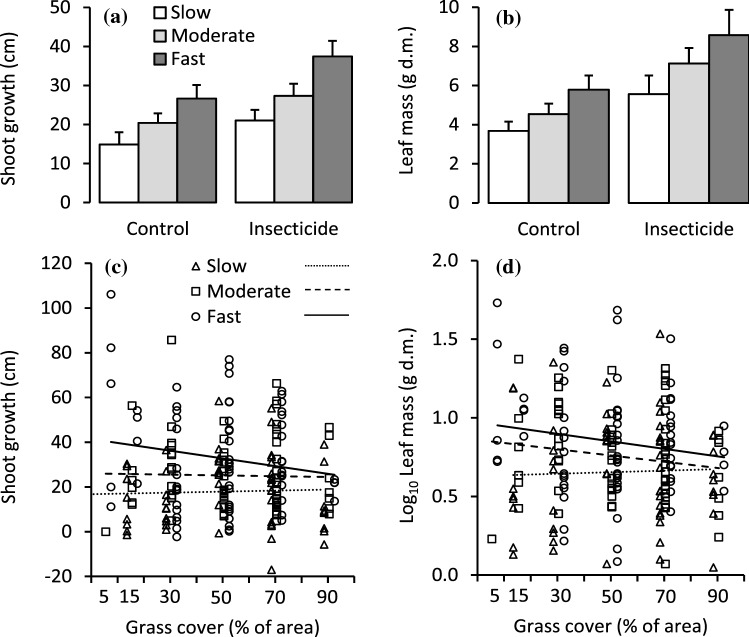
Shoot growth and leaf mass responses in silver birch growth classes to **a**, **b** insecticide treatment (mean + SE, *n* = 28–38) and **c**, **d** grass cover (*n* = 56–74). In **b** the means and errors are back transformed from log-transformed data; in **c** and **d** the lines are linear regressions

The growth class × insecticide interaction effect was not statistically significant for either shoot growth or leaf mass (Table 1, Fig. 2a, b). Instead, the insecticide main effect explained 4.7 and 8.3% of total shoot growth and leaf mass variation and increased shoot and leaf growth on average by 38 and 52%, respectively (Table 1, Fig. 2a, b). The fast-growing genotypes grew on average 78% more and produced 56% more leaf mass than the slow-growing genotypes (Table 1, Fig. 2a, b). Within growth classes, the genotype did not significantly affect shoot growth or leaf mass (Table 1). The growth class × grass cover interaction effect explained 3.5 and 2.6% of total shoot growth and leaf mass variation and was significant for both (Table 1). This was because shoot and leaf growth of fast-growing, but not of slow-growing genotypes was negatively affected by increasing grass cover (Fig. 2c, d). Another interpretation for the interaction is that sapling growth differed between the fast-growing and slow-growing genotypes in low but not in high grass cover (Fig. 2c, d).

**Table 1:** The effects of silver birch growth class (groups of genotypes with slow, medium and fast growth), genotype (nested within growth class), insecticide treatment (treatment and control) and planting plot grass cover (a continuous variable) on silver birch sapling attributes as tested using ANCOVA

Response variable	Growth class (GC)			Genotype			Insecticide			Grass cover			GC × insecticide			GC × grass cover		
	*F*	*P*	*R*^2^	*F*	*P*	*R*^2^	*F*	*P*	*R*^2^	*F*	*P*	*R*^2^	*F*	*P*	*R*^2^	*F*	*P*	*R*^2^
Shoot growth	10.2	< 0.001	7.3	0.47	0.949	2.5	12.1	0.001	4.7	< 0.01	0.993	< 0.1	0.41	0.663	0.3	4.53	0.012	3.5
Leaf mass	6.59	0.002	4.9	1.09	0.367	5.6	22.8	< 0.001	8.3	1.19	0.276	0.4	0.19	0.827	0.1	3.57	0.030	2.6
Leaf N%	2.83	0.063	2.9	1.62	0.078	11	4.65	0.033	2.2	0.26	0.611	0.1	1.41	0.246	1.3	1.19	0.308	1.1
Leaf damage	2.11	0.124	1.1	0.60	0.863	2.2	177	< 0.001	47	0.17	0.684	< 0.1	0.24	0.787	0.1	0.74	0.480	0.4
Uptake of added N	3.97	0.021	3.5	1.12	0.348	6.8	12.4	0.001	5.4	< 0.01	0.979	< 0.1	0.71	0.494	0.6	1.97	0.143	1.7
Root mass	0.89	0.413	1.8	1.21	0.291	16	0.61	0.438	0.6	7.64	0.007	7.4	1.58	0.214	3.1	0.85	0.432	1.7
Root allocation index	0.46	0.636	0.9	0.90	0.558	13	0.14	0.710	0.1	8.57	0.005	8.7	1.16	0.320	2.4	0.37	0.693	0.7
Ergosterol mass	3.93	0.022	2.6	0.86	0.602	4.0	0.53	0.469	0.2	1.91	0.169	0.6	0.56	0.570	0.4	3.06	0.049	2.0

Field replicate block, top shoot damage and study year were included in the models when applicable but are not reported in the table

**Fig. 3:**
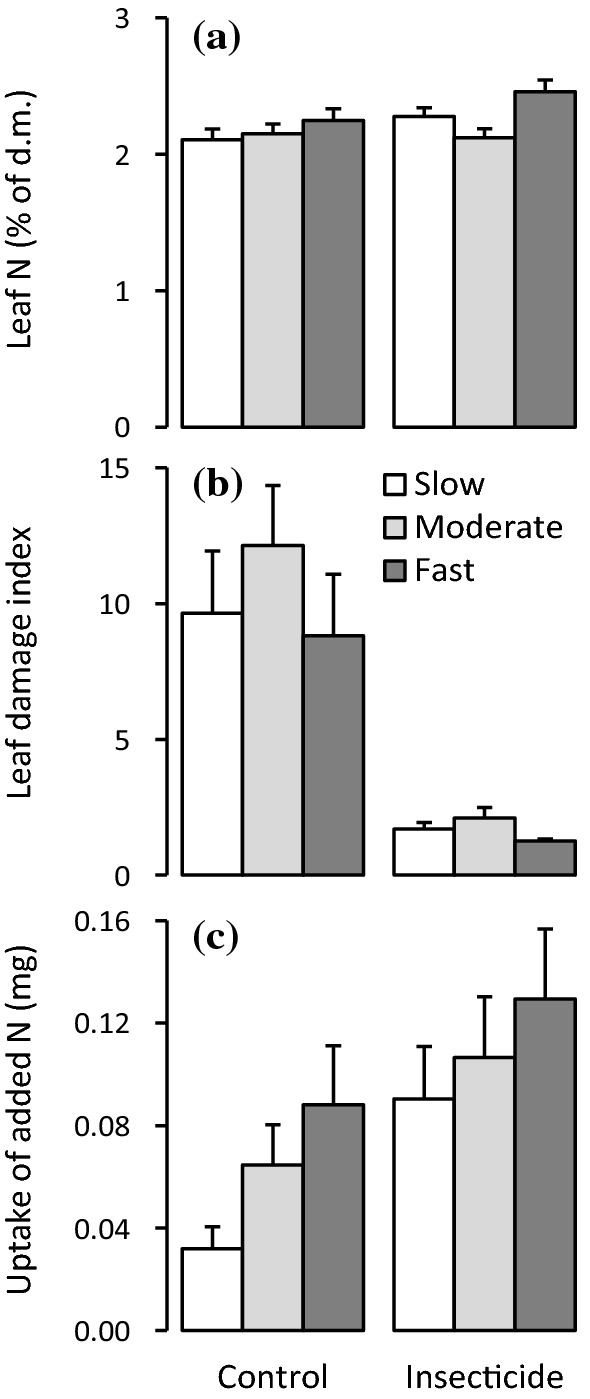
Responses of **a** leaf N concentration, **b** leaf damage by herbivores and **c** soil N uptake in silver birch growth classes to insecticide treatment (mean + SE; *n* = 27–38). In **b** and **c** the means and errors are back transformed from log and square-root-transformed data, respectively

The growth class × insecticide and growth class × grass cover interaction effects were not statistically significant for leaf N concentration, leaf damage and leaf N uptake (Table 1). Instead, the insecticide main effect explained 2.2 and 5.4% of total leaf N concentration and N uptake variation and increased them by 5.4% and 76%, respectively (Table 1, Fig. 3a, c). Leaf damage was on average 83% lower for̄ the insecticide treated than control saplings (Table 1, Fig. 3b). Leaf N uptake was 78% higher in fast-growing than slow-growing genotypes, but leaf damage and N concentration did not differ among the growth classes (Table 1, Fig. 3a–c). Grass cover explained ≤ 0.1% of total variation in these three variables and had no significant effect on any (Table 1).

**Fig. 4:**
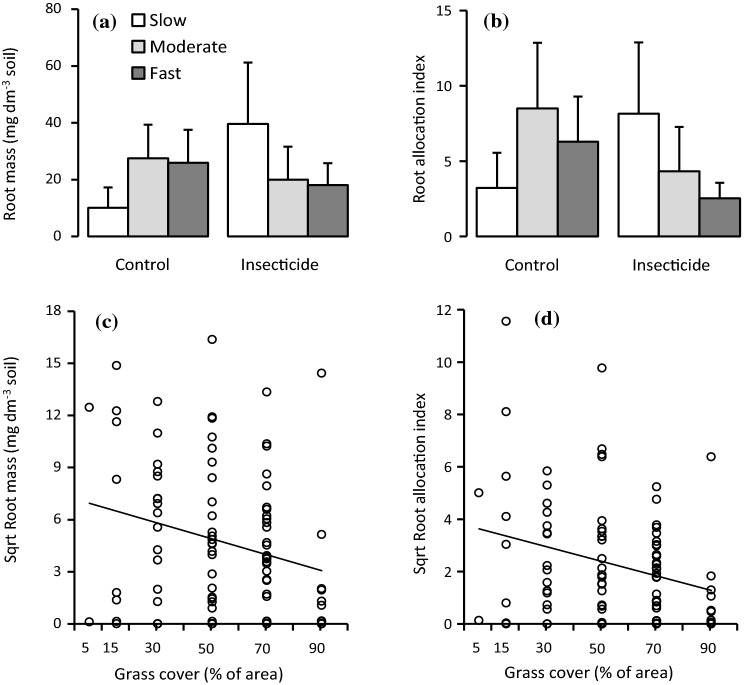
Responses of silver birch root mass and root allocation index (index of leaf mass-root mass ratio) to **a**, **b** insecticide treatment (mean + SE, *n* = 13–19) and **c**, **d** grass cover (*n* = 97). In **a** and **b** the growth class means and errors are back transformed from square-root-transformed data; in **c** and **d** the lines are linear regressions that describe the general response across all growth classes as no growth class × grass cover interaction effect was found

Root mass and the index of the root mass–leaf mass ratio were not affected by growth class × insecticide and growth class × grass cover interactions (Table 1). Instead, both decreased significantly with increasing grass cover, which explained 7.4 and 8.7% of their total variation, respectively (Table 1, Fig. 4c, d). Root mass and the index of the root mass–leaf mass ratio did not differ among growth classes and neither was there statistically significant genetic variation within the classes, but the proportion of total variation explained by genotype was high, 16 and 13%, respectively (Table 1, Fig. 4a, b). The insecticide treatment had no effect on either variable (Table 1, Fig. 4a, b).

**Fig. 5:**
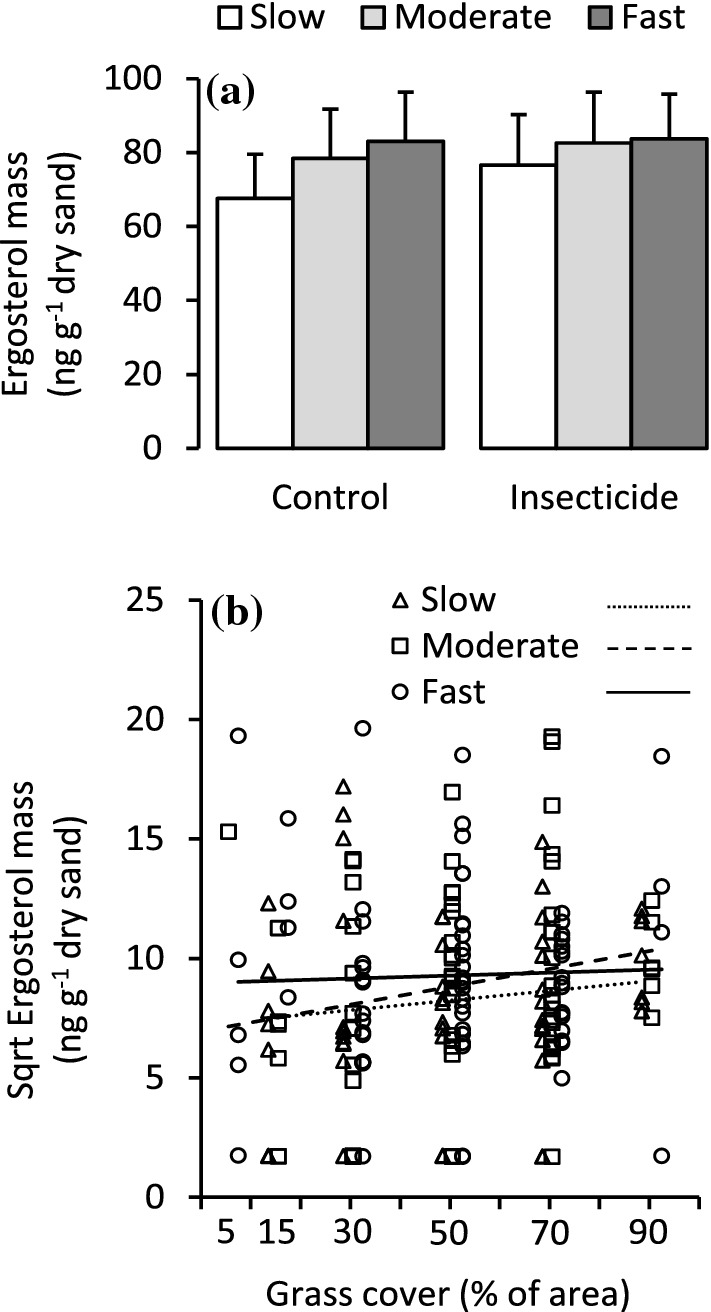
Response of quartz sand ergosterol mass in silver birch growth classes to **a** insecticide treatment (mean + SE, *n* = 27–36) and **b** grass cover (*n* = 55–72). In **a** the means and errors are back transformed from square-root-transformed data; in **b** the lines are linear regressions

In contrast to root mass, the growth class × grass cover interaction effect was significant for ergosterol mass, explaining 2.0% of total variation (Table 1). This was because ergosterol production responded positively to increasing grass cover in the slow and moderate growth class, but not in the fast growth class (Fig. 5b). Ergosterol mass was on average 16% higher in the fast-growing than slow-growing genotypes (Table 1, Fig. 5a), but the significant growth class × grass cover interaction effect suggests that the difference existed in low grass cover only (Table 1, Fig. 5b). The insecticide treatment had no effect on ergosterol mass (Table 1, Fig. 5a).

## Discussion

### Traits associated to fast growth

We predicted that the silver birch genotypes with fast and slow growth would exhibit the typical traits of fast-growing and slow-growing plant species (Wright et al. 2004; Reich 2014; De Deyn 2017), with fast-growing genotypes (defined by their shoot elongation) having better capture of soil N, higher leaf N concentration and greater production of leaves and roots. In line with these predictions, the fast-growing genotypes transferred more N from soil to their leaves and produced more leaves, but did not produce more root biomass or have higher leaf N concentration. We further predicted that as a sign of higher investment to aboveground than belowground growth, the fast-growing genotypes would have lower root mass–leaf mass ratio and would support lower mass of mycorrhizal hyphae. In contrast to these predictions, there was no significant difference in relative root allocation among the growth classes (but instead a large share of genetic variation remained within growth classes) and the fast-growing genotypes maintained higher abundance of EM fungal hyphae in plots with low grass cover.

These results suggest that among the silver birch genotypes the fast growth (and the high C acquisition it requires) is not governed by higher leaf N concentration, which is typically found when fast-growing and slow-growing species are contrasted (Wright et al. 2004), but simply by better capture of mineral N and greater production of leaves. Moreover, while the fast-growing species are suggested to allocate less resources to belowground mutualists (De Deyn 2017), our results suggest that the better capture of soil mineral N by fast-growing genotypes was based on resource allocation to mycorrhizae rather than to roots (but importantly, our results only tell of total root biomass, not architecture or activity). This is in good agreement with the findings that EM association is highly beneficial for the growth of silver birch (Frankland and Harrison 1985) despite its fast-growing, early-successional character. In a comparison of annual and perennial plants colonized by AM fungi, Roumet et al. (2006) also found that the roots of fast-growing annuals had higher mycorrhizal colonization than the roots of perennials. Together all these results seem to suggest that investing to mutualism with mycorrhizal fungi may be a key ‘root trait’ for fast-growing genotypes to maintain the efficient belowground nutrient acquisition needed for fast aboveground biomass production.

### Tradeoffs of fast growth

We predicted that the tradeoffs of fast growth found among plant species (Coley et al. 1985; Herms and Mattson 1992; Rees et al. 2001) would also emerge among silver birch genotypes, with shoot elongation, leaf production and soil N capture being more adversely affected by insect herbivory and resource competition for fast-growing than for slow-growing genotypes. Herbivory had a very significant, negative impact on sapling shoot growth, leaf production and leaf N uptake, and also a minor negative impact on leaf N concentration, but did not have a measurable effect on root mass, index of root mass–leaf mass ratio or mycorrhizal fungal production. In contrast to what we expected, and despite the effects of herbivory on shoot production being severe, there was no sign of growth class × insecticide interaction effect on either plant growth or soil N acquisition, i.e. fast growers were not more susceptible to herbivory than slow growers. This is further supported by our finding that leaf damage did not differ between fast and slow growers and indicates no genotypic tradeoff between growth rate and herbivore resistance in our population.

However, a growth rate-herbivore resistance tradeoff has earlier been found in the same local silver birch population by Pusenius et al. (2002), with insects and voles preferring fast-growing over slow-growing families. The seedlings used by Pusenius et al. (2002) were produced from freely pollinated seeds (i.e. the “genotypes” consisted of families of half-siblings) in contrast to our saplings that were vegetatively propagated from buds (the genotypes consisted of genetically identical individuals). Since the seeds and buds originate from the same mother trees, originally selected for measuring the variation of defense chemistry within the population (Laitinen et al. 2000), it is unlikely that a genetic growth rate-herbivore resistance tradeoff would be present in one plant material, but absent in another. It is more likely that the discrepancy between the results originates from differences in the experimental set-up. Pusenius et al. (2002) produced dense seedling patches with no other plants and seedlings only 5 cm apart, whereas our saplings were planted amongst natural ground layer vegetation 2 m apart. Although in both cases, the genotypes were randomly distributed among each other, it is likely that from the herbivore’s perspective comparing the genotypes and preferring one over another is easier in a dense patch of seedlings than among sparsely distributed plants. This reasoning would imply that our silver birch population indeed has a genetic tradeoff between growth rate and herbivore resistance, but the appearance of the tradeoff depends on the environmental context. As patches of young birch seedlings can be very dense (> 700 individuals m^−2^) at open, disturbed soil patches (Kinnaird 1974), but also sparsely distributed seedlings are common, neither our nor the Pusenius et al. (2002) set-up can be argued to be artificial. This echoes the view that detecting tradeoffs is anything but straightforward (Koricheva 2002; Agrawal 2011).

Increasing field grass cover had a negative effect on both shoot and root production of birch saplings, thus implying intensifying resource competition with increasing grass abundance. Supporting our prediction of a tradeoff between fast growth and competitive ability, shoot growth and leaf production of fast-growing genotypes were on average 42% and 25% lower, respectively, in plots of highest grass cover than in plots of lowest grass cover, whereas the slow-growing genotypes expressed a 16% and 8% increase of shoot and leaf production, respectively, in the same gradient. In contrast to shoot elongation and leaf growth, root mass response to increasing grass cover did not vary among the growth classes, but was generally 49% lower in the highest than lowest grass cover class. This and the equally decreasing index of root mass–leaf mass ratio show that birch saplings responded to increasing grass competition by allocating relatively more resources to shoot production, which in turn suggests that competition with grasses was more for light than for nutrients. This makes sense as the grass *C. arundinacea* that dominated the ground layer vegetation at our site can grow up to 150 cm tall and the mean height of saplings in spring 2011 was 50 cm. On the other hand, it is good to note that at least *D. flexuosa* is also suspected to allelopathically suppress tree root growth (Jarvis 1964). When we were earlier following the growth of our saplings, we found a clear G × E interaction between the growth in benign nursery conditions and the growth in harsher field conditions (Mikola et al. 2014). The present results seem to tell the same story: the genotypes that grow fast in favorable conditions lose their lead in harsher conditions. While this is a clear evidence of growth rate-competitive ability tradeoff, how were the slow-growing genotypes able to maintain their shoot production under increasing competition?

The answer seems to hide in the mycorrhizae. Our results show that increasing grass abundance induced greater production of EM fungal hyphae, but only in genotypes with slow and moderate growth. In these genotypes, production of hyphae was on average 49% higher in plots with highest than in plots with lowest grass cover. This upsurge of hyphal production equates the reduction of root biomass along the grass cover gradient, which suggests that slow-growing genotypes compensated for the reduction in root production by allocating more resources to mycorrhizal fungal production. This would allow acquiring more nutrients to support the aboveground growth and indeed, is an indication of soil mutualists being a part of slow growth strategy as suggested by De Deyn (2017). In fact, if we calculate relative resource allocation to mycorrhizae as an ergosterol mass–leaf mass ratio (ng ergosterol g^−1^ dry sand per g leaf dry mass), the slow-growing genotypes seemed to allocate relatively more resources to mycorrhizae than fast-growing genotypes, the ratio being on average 43.4 ± 15.0 (mean ± SE), 36.5 ± 6.8 and 25.6 ± 4.1 for genotypes of slow, moderate and fast growth, respectively. Although these differences are not statistically significant (*P* = 0.242 for the slow growth vs. fast growth comparison), together with the finding of increasing hyphae production by slow-growing genotypes in response to increasing grass cover they seem to suggest that mycorrhizal fungi may have a twofold role in the growth of fast and slow genotypes. The higher mycorrhizal fungal production of fast-growing genotypes in our study plots with low grass cover may be a signal of the role of mycorrhizal fungi in acquiring nutrients for the benefit of fast growth. The higher relative allocation of resources to mycorrhizal fungi and the positive response of this allocation to increasing competition in slow-growing genotypes may in turn be a signal of the role of mycorrhizae in the better competitiveness of slow growers. The latter reasoning seems to be supported by a recent finding that colonization of plant roots by AM fungi increased the competitive ability of weak competitors and reduced the magnitude of variation in competitive ability among species in an old-field herbaceous plant community (Stanescu and Maherali 2017). On the other hand, it is good to realize that looking at fungal growth only may hide important variation in the community structure of fungi. This variation could be as important as the variation in growth as shown for *Pinus edulis* genotypes, whose differences in drought tolerance seem to stem from the differences in the structure of their EM fungal communities (Gehring et al. 2017). Finally, to put our results into a wider perspective, Ostonen et al. (2017) showed that production of absorptive fine roots and EM external mycelium increased per tree basal area in silver birch with increasing soil C:N ratio (indicating decreasing soil N availability) along a latitudinal gradient from 48°N to 69°N. This is a clear indication of soil nutrient deficiency leading not only to slower growth, but also to relatively greater resource allocation to root and mycorrhizal fungal production. Although part of this latitudinal variation likely stems from phenotypic plasticity, these results suggest that at the interpopulation scale, silver birch growth rate and the relative allocation of resources to the EM association have a negative genetic association.

## Conclusions

We have recently shown that a tradeoff between efficient N resorption in senescing leaves and subsequent soil N availability, which is typically discovered in interspecific studies (Aerts 1997), has a genotypic counterpart in our silver birch population (Mikola et al. 2014). The present study further suggests that fast growth and competitive ability have a genotypic intrapopulation tradeoff. These findings suggest that genotypic tradeoffs have a significant role in controlling the evolution of traits and maintaining their genetic variation in silver birch populations. As a novelty, our results suggest that mycorrhizal fungi may have a key role in supporting the N acquisition of fast-growing silver birch genotypes in less competitive environments, but also in explaining the fast growth-competitive ability tradeoff and the relatively better performance of slow-growing genotypes in more competitive environments. Whether these findings are common in plant populations and whether the mycorrhizal fungi generally play a significant role in the arena of tradeoffs of plant traits needs to be verified in future investigations.

## Supplementary Information

Below is the link to the electronic supplementary material.

Supplementary file1 (PDF 633 KB)

## Data Availability

Data available in Mikola, Juha et al. (2021) Data from: Genotypic traits and tradeoffs of fast growth in silver birch, a pioneer tree. Dryad, Dataset, 10.5061/dryad.0p2ngf21w.
