# Supplementary material for: Genotypic traits and tradeoffs of fast growth in silver birch, a pioneer tree
Source: Oecologia. 2021 Jul 26;196(4):1049–60. doi: 10.1007/s00442-021-04986-9 (PMC8367902; doi:10.1007/s00442-021-04986-9)
Supplement: Supplementary file 1 — Supplementary file1 (PDF 633 KB) [file 442_2021_4986_MOESM1_ESM.pdf]

**Supplementary Information for:**

Oecologia

# Genotypic traits and tradeoffs of fast growth in silver birch, a pioneer tree

Juha Mikola, Katariina Koikkalainen, Mira Rasehorn, Tarja Silfver, Ulla Paaso, Matti Rousi

**Corresponding author:**
Juha Mikola
Group manager, Carbon cycle management
Department of Bioeconomy and environment
Natural Resources Institute Finland (Luke)
Latokartanonkaari 9
00790 Helsinki
Finland

Email: juha.mikola@luke.fi
Tel. +358503082364

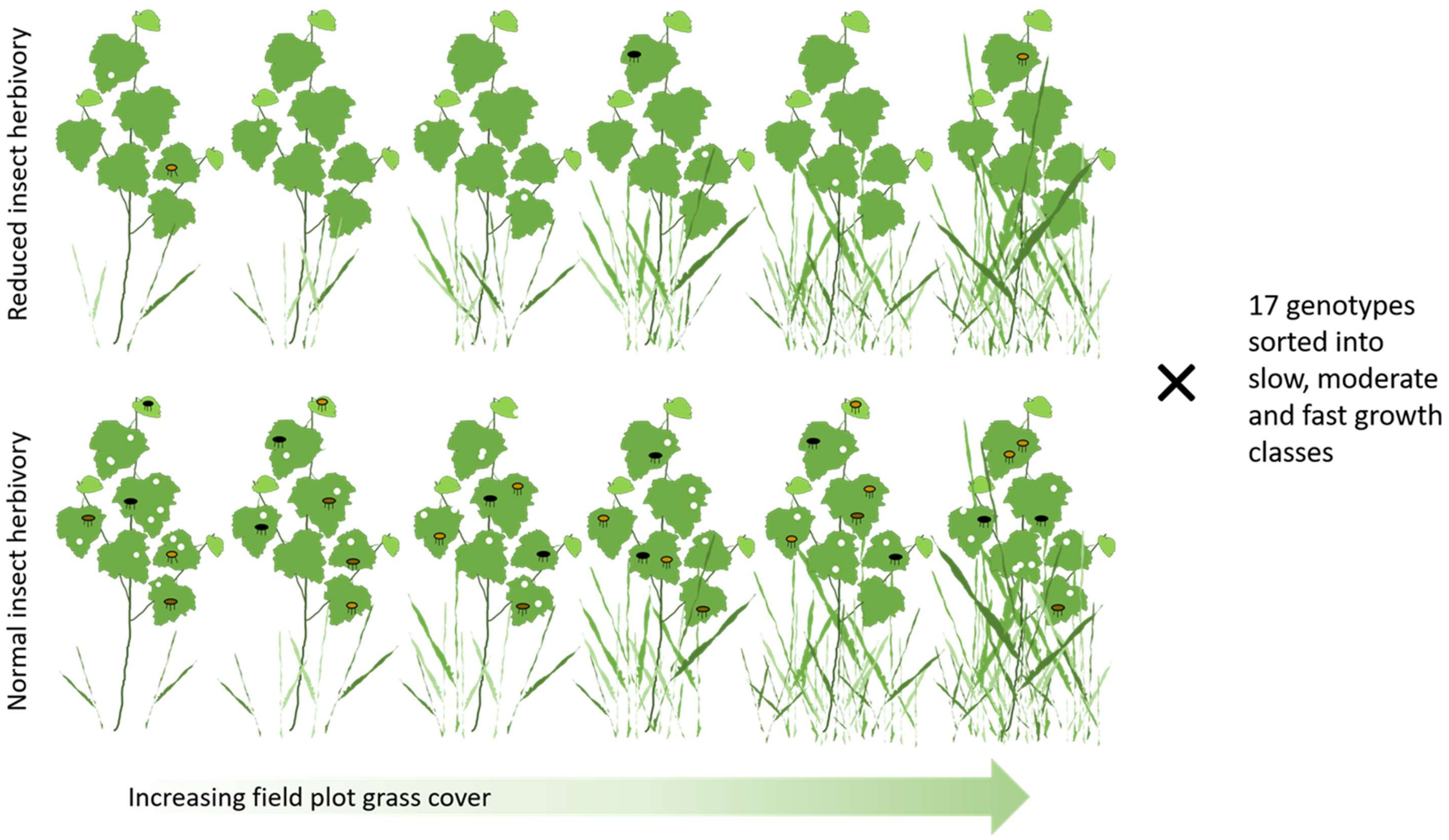


**Supplementary Figure 1.** In the two-year field experiment, the twelve saplings of each of the 17 silver birch genotypes were subjected to both the insect herbivory treatment (reduced insect herbivory achieved using weekly insecticide sprayings) and the natural gradient of field plot grass cover (ranging from 5% to 90%). Based on the shoot growth rate, the genotypes were further sorted into slow, moderate and fast growth classes.
